# Research Progress on Angiogenesis and Involution Mechanisms of Infantile Hemangioma and Its Regulation by Active Components of *Salvia miltiorrhiza*

**DOI:** 10.3390/ijms27177841

**Published:** 2026-09-02

**Authors:** Yuan Lu, Yamin Wu, Nan Gu, Ke Wen, Chao Yang, Xuetao Huang, Dedong Zeng, Lu Huang, Shimin Wu, Shumin Ma, Qian Liu

**Affiliations:** 1 Integrated Chinese and Western Medicine Institute for Children Health & Drug Innovation, Discipline of Chinese and Western Integrative Medicine, Jiangxi University of Chinese Medicine, Nanchang 330004, China; luluyuanyuan1995@163.com (Y.L.);; 2 Centre for Translational Medicine, College of Traditional Chinese Medicine, Jiangxi University of Chinese Medicine, Nanchang 330004, China

**Keywords:** infantile hemangioma, angiogenesis, involution mechanisms, active components of *Salvia miltiorrhiza*, regulatory mechanisms

## Abstract

Infantile hemangioma (IH) is the most common benign vascular tumor of infancy, characterized by a unique life cycle encompassing a rapid proliferative phase followed by spontaneous involution. Although the majority of cases exhibit a favorable prognosis, a small subset may be complicated by severe sequelae that adversely affect the physical and mental health of affected infants. Accumulating evidence indicates that the pathogenesis of IH involves a dynamic imbalance between angiogenesis and involution, which is tightly governed by the multilineage differentiation of hemangioma stem cells, core signaling pathway networks, and epigenetic regulation mediated by non-coding RNAs. A deeper understanding of the mechanisms underlying angiogenesis and involution will provide novel insights into both the fundamental biology and clinical management of IH. Furthermore, multiple bioactive constituents of the traditional Chinese medicine *Salvia miltiorrhiza* (Danshen) have demonstrated anti-angiogenic and pro-involution potential in tumor and ischemia models. This review systematically summarizes recent advances in the mechanistic understanding of IH angiogenesis and involution, and critically discusses the regulatory effects of Danshen active components on these processes, with the aim of offering a theoretical foundation for elucidating IH pathogenesis and facilitating the development of novel therapeutic agents.

## 1. Introduction

Infantile hemangioma (IH) is a common benign vascular tumor of infancy and early childhood, predominantly involving the head, face, and neck. It exhibits a distinctive clinical course of rapid proliferation followed by slow spontaneous involution [1], affecting approximately 5% of newborns, with an estimated incidence of 2–10% [2]. Although most lesions regress naturally over time, about 10–15% may give rise to severe complications, including ulceration, bleeding, visual impairment, and airway obstruction. In some cases, residual scarring, pigmentary changes, and fibrofatty tissue overgrowth can occur, profoundly affecting the physical appearance and psychosocial well-being of affected children. The core pathological hallmark of IH lies in the uncontrolled aberrant angiogenesis and imbalanced programmed involution, driven by excessive endothelial cell proliferation and disorganized vascular remodeling. Propranolol, although currently the first-line clinical therapy for IH, has its application constrained by potential adverse effects and an incompletely understood molecular mechanism [3,4]. Recent research has revealed that the anti-angiogenic effect of this drug is not mediated via the classical β-adrenergic receptor, but instead through an off-target action of its R(+) enantiomer targeting the endothelial transcription factor SOX18 [5]. This finding not only overturns conventional views, but also points to a much more complex regulatory network of IH angiogenesis than previously recognized. Therefore, a comprehensive dissection of the fine-tuned regulatory networks governing IH angiogenesis is of great significance for identifying novel therapeutic targets and optimizing treatment strategies. Recent studies have revealed that IH angiogenesis and involution are precisely orchestrated by multi-cellular interactions, multiple signaling pathways, and microenvironmental cues. Notably, the roles of non-coding RNA regulatory networks, metabolic reprogramming, and epigenetic modifications in determining IH have become increasingly prominent [6,7,8,9]. Furthermore, multiple active components from the traditional Chinese medicine *Salvia miltiorrhiza* (Danshen) demonstrate significant pharmacological actions, including anti-angiogenesis, anti-inflammation, anti-oxidation, and inhibition of tumor growth [10]. This review systematically summarizes recent advances in the mechanisms underlying IH angiogenesis, involution, and microenvironmental modulating factors, with a particular emphasis on the vascular regulatory effects and molecular actions of bioactive constituents from Danshen. The aim is to provide a theoretical foundation for elucidating IH pathogenesis, facilitating targeted therapy with traditional Chinese medicine, and guiding the development of novel clinical drugs.

## 2. Review Methodology

This review was conducted as a narrative review. Data for this review were identified via searches within PubMed, Web of Science, and references from relevant articles. In PubMed, Medical Subject Headings (MeSH) and keywords paired with Boolean connectors (AND) were used to retrieve articles. The following keyword combinations were used for the PubMed database: (“infantile hemangioma” OR “hemangioma”) AND (“angiogenesis” OR “involution” OR “regression”), (“Salvia miltiorrhiza” OR “tanshinone” OR “salvianolic acid”) AND (“hemangioma”), (“propranolol”) AND (“mechanism”), and (“vascular endothelial growth factor”) AND (“hemangioma”). For the Web of Science database, the searches were performed using “infantile hemangioma” and “Salvia miltiorrhiza” separately. We selected studies based on their relevance to the topic, without applying a formal systematic review framework or quantitative quality assessment. The inclusion criteria involved articles that reported on angiogenesis, regression mechanisms of infantile hemangioma, or the regulatory effects of active components from Salvia miltiorrhiza, including in vitro, in vivo, or ex vivo experimental studies, original research articles, and articles written in English and published between 1992 and 2026, with particular emphasis on those published in the past 10 years. However, we excluded conference proceedings and articles on diseases unrelated to infantile hemangioma or active components of Salvia miltiorrhiza. The extracted information included experimental models (cell lines or animal models), drug concentrations, involved signaling pathways, angiogenesis-related factors, regression mechanisms, efficacy, and mechanisms of action.

## 3. Mechanisms of Angiogenesis and Involution

Angiogenesis in IH is a dynamic pathological process coordinately regulated by multiple cell types and signaling pathways, the crux of which lies in an imbalance between vasculogenesis and angiogenesis [11]. Specifically, vasculogenesis denotes the in situ differentiation and assembly of endothelial precursor cells into primitive vascular networks, whereas angiogenesis refers to the sprouting of new vessels from pre-existing vessel walls—a process that serves as the predominant mechanism driving rapid vascular expansion during the proliferative phase of IH [12]. During proliferation, hyperactivation of VEGFA/VEGFR2 signaling and the hypoxia-HIF-1α-VEGFA positive feedback loop constitute the central drivers of aberrant vascular overgrowth [13,14]. In contrast to these proliferative mechanisms, involution is characterized by a shift in cellular fate and suppression of pro-angiogenic signals. At the cellular level, hemangioma stem cells (HemSCs) switch from endothelial to adipogenic differentiation, leading to replacement of vascular structures by fibrofatty tissue [15,16]. At the signaling level, activities of pathways such as PDGF and Notch are dampened, whereas adipogenic and anti-angiogenic factors are upregulated [17]. At the microenvironmental level, infiltration of M1-type macrophages induces endothelial-to-mesenchymal transition (EndMT), further facilitating involution. These proliferative and involutive mechanisms exhibit dynamic opposition and synergy in terms of cellular fate and signaling pathways, collectively shaping the unique life cycle of IH from rapid growth to spontaneous regression, which is precisely governed by the multilineage differentiation of HemSCs, signaling networks, and epigenetic regulation [18,19].

### 3.1. Multilineage Differentiation of HemSCs

**Figure 1:**
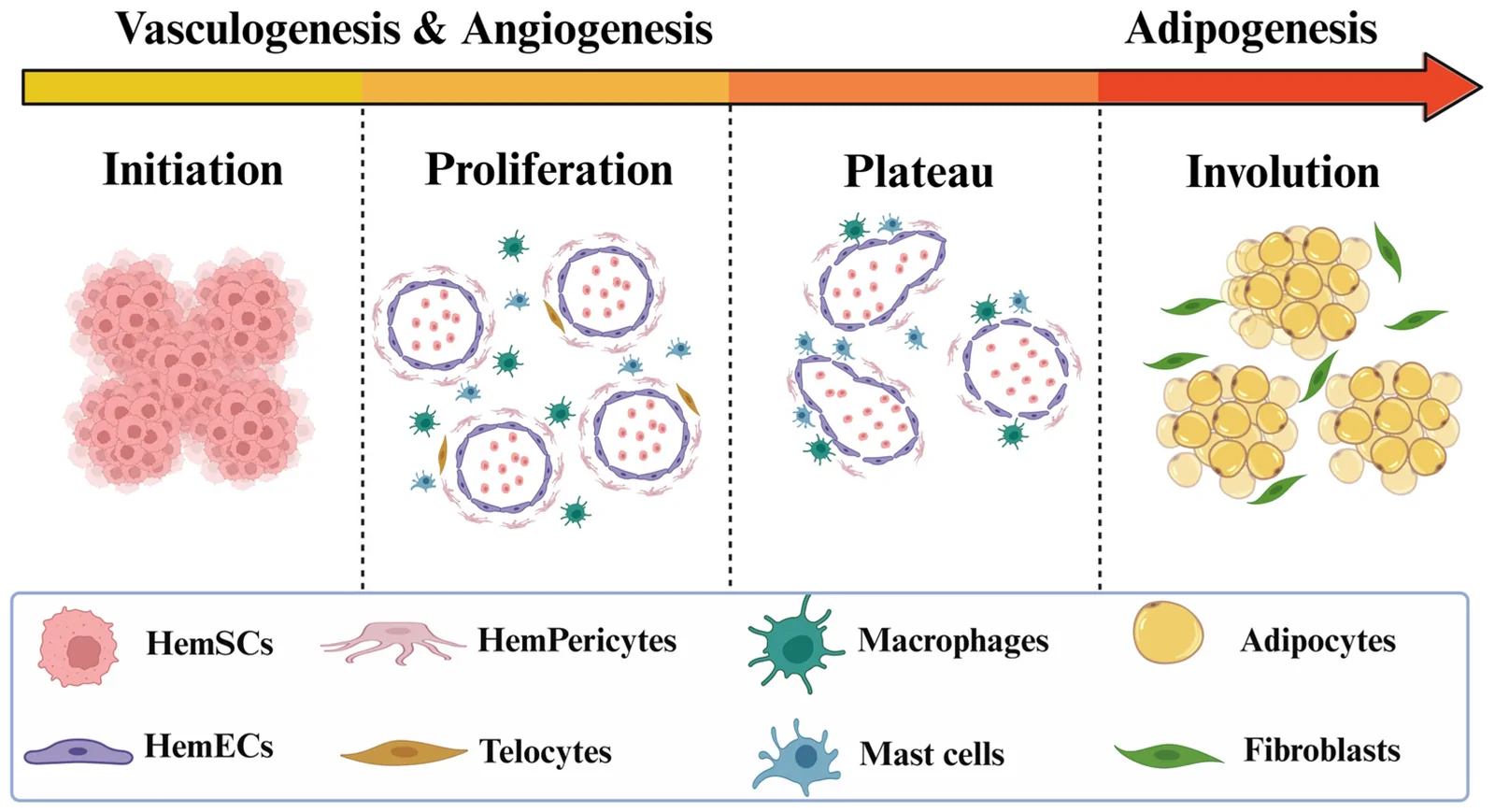
Cellular compositions of IH at different clinical stages. Created in BioRender. Lu, Y. (2026) https://BioRender.com/giu7weg.

The histological architecture of IH is complex, encompassing multiple cell types including HemSCs, progenitor cells, endothelial cells (HemECs), pericytes (HemPCs), adipocytes, fibroblasts, and telocytes. Recent advances in single-cell RNA sequencing (scRNA-seq) have further uncovered previously unappreciated cellular heterogeneity within IH tissues. Notably, mural cells have been identified as the most abundant cell population in IH, constituting >50% of all cells, and can promote angiogenesis by secreting ligands such as VEGFA and PGF that act on endothelial cells [20]. Among these diverse cell types, HemSCs are widely considered to be one of the core cellular sources driving both vasculogenesis and involution, possessing multilineage differentiation potential. During the proliferative phase, HemSCs predominantly differentiate into HemECs and HemPCs, fueling the construction of aberrant vascular networks. During involution, the same stem cells transdifferentiate into adipocytes, resulting in reduced vascular components and replacement by fibrofatty tissue (Figure 1). In this process, Krüppel-like factor 2 (KLF2) serves as a key transcriptional regulator that governs the fate determination of HemSCs. During the proliferative phase, its elevated expression drives HemSCs toward endothelial differentiation. In the involution phase, its expression wanes, which relieves the predominant effect on endothelial commitment and indirectly facilitates adipocyte formation [21]. Additionally, the m6A methylation-related protein KIAA1429 is highly expressed in involuting IH, where it induces HemECs to adopt a facultative stem cell state in a methylation-independent manner. This conversion enhances their stemness and promotes transdifferentiation toward a fibroblast-like phenotype, thereby driving IH involution [22]. These findings indicate that the proliferation, differentiation, and transdifferentiation of HemSCs are finely tuned by transcription factors and epigenetic modifiers, which often exert their effects through activation or inhibition of downstream core signaling pathways.

### 3.2. Networked Regulation by Core Signaling Pathways

**Figure 2:**
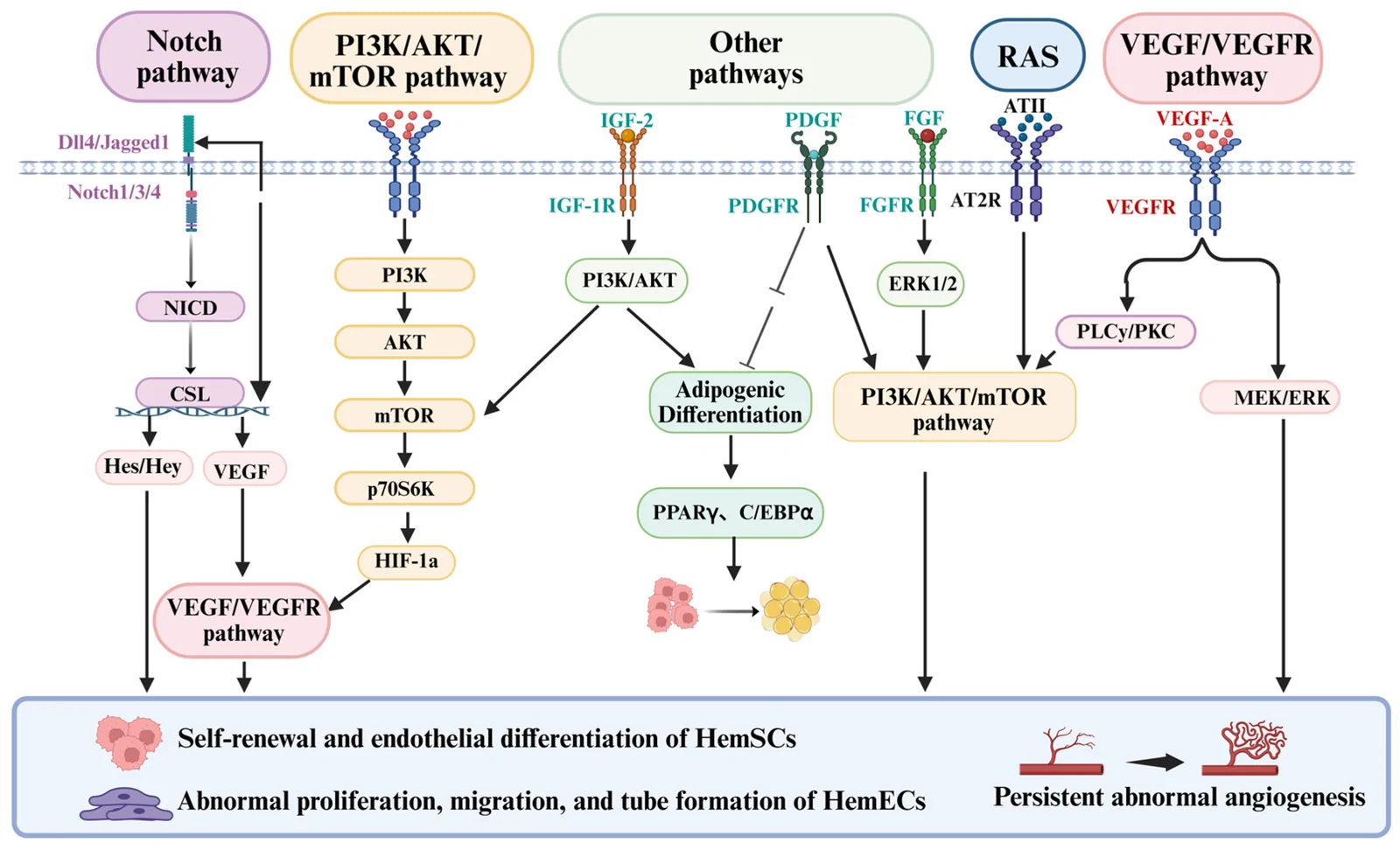
Core signaling pathway network regulating IH angiogenesis and involution. Created in BioRender. Lu, Y. (2026) https://BioRender.com/cu9riy8.

The proliferation and involution of IH are not driven by any single signaling pathway independently, but rather by a networked interplay of multiple pathways that cross-talk and dynamically switch among different cell types and stages (Figure 2). During the proliferative phase, the VEGF/VEGFR, PI3K/AKT/mTOR, and Notch pathways are highly activated, collectively promoting abnormal proliferation, migration, and tube formation of HemECs, while maintaining HemSCs’ self-renewal and endothelial differentiation potential. Upon entering the involution phase, the activities of the aforementioned pro-angiogenic pathways are gradually suppressed. Concurrently, the expression of adipogenic differentiation-related signals, such as PPAR-γ and C/EBPα/β, along with anti-angiogenic factors, becomes upregulated, which drives HemSCs toward adipogenic differentiation. Moreover, the PDGF/PDGFR pathway acts as a negative regulator of involution, inhibiting adipogenesis during proliferation, whereas its blockade accelerates regression. This dynamic opposition and synergy between the activation of pro-angiogenic pathways and the suppression of pro-involution signals forms the signaling network foundation for the transition from rapid proliferation to spontaneous involution in IH. The regulatory mechanisms of each major signaling pathway in IH are elaborated below.

#### 3.2.1. VEGF/VEGFR Signaling Pathway

The VEGF/VEGFR axis serves as the central hub governing angiogenesis in IH. The human VEGF family includes VEGF-A, VEGF-B, VEGF-C, VEGF-D, and placental growth factor, with receptors VEGFR-1 and VEGFR-2 belonging to the tyrosine kinase family [23]. VEGF-A is the critical pro-angiogenic factor [24]. In HemECs, defective VEGFR/integrin complexes lead to reduced expression of VEGF “decoy” receptors, resulting in constitutive activation of VEGFR2 signaling [25]. HemECs highly express VEGFR-2, and specific binding of VEGF-A to this receptor potently activates multiple downstream cascades, including PI3K/AKT, MEK/ERK, and PLCγ/PKC, eliciting a cascade reaction [26]. Meanwhile, HemECs can sustain VEGF-A/VEGFR-2 signaling through an autocrine loop, which promotes their own proliferation, migration, and survival. Moreover, when VEGFR-1 is expressed at low levels in HemECs, the downregulation of this receptor further enhances VEGFR-2 signaling and exacerbates aberrant angiogenesis, as VEGFR-1 competes with VEGF-A for binding [27]. Clinical data confirm that serum VEGF levels in children with proliferating IH are significantly higher than those in involuting patients and healthy controls [28], underscoring the driving role of VEGF in IH proliferation.

#### 3.2.2. PI3K/AKT/mTOR Signaling Pathway

Angiogenesis in IH is a complex pathological process synergistically regulated by multiple pathways. Beyond the core VEGF/VEGFR axis, several other signaling cascades are aberrantly activated and collectively contribute to vascular formation and disease progression. Among them, the PI3K/AKT/mTOR pathway is a central node in IH aberrant angiogenesis. This pathway is hyperactivated in HemECs, characterized by phosphorylated PI3K activating downstream AKT (including isoforms AKT1, AKT2, AKT3) [29], which in turn activates mTOR and its downstream p70S6K cascade. Activation of this pathway promotes endothelial cell hyperproliferation and migration [30]. Notably, this pathway represents a critical convergence point for IH angiogenic signals. By examining the role and molecular mechanisms of AKT/mTOR in IH, it was found that pathway activity exhibits a dynamic decline from proliferation to involution. Combined blockade of AKT/mTOR downregulates PCNA expression, suppresses proliferation, and induces apoptosis [31]. Furthermore, Ke et al. [32] demonstrated that the deubiquitinase USP18 is highly expressed in IH and promotes HemEC growth and inhibits apoptosis by enhancing AKT phosphorylation. Similarly, Ren et al. [33] revealed that STC2 promotes HemEC proliferation and angiogenesis through activating the VEGFR2/AKT/eNOS pathway. Collectively, these findings establish the PI3K/AKT/mTOR pathway and its upstream/downstream regulatory network as a core hub in IH angiogenesis.

#### 3.2.3. Notch Signaling Pathway

Beyond the classic pro-angiogenic pathways, the Notch pathway also plays a critical role in vascular development and pathological neovascularization. The core functional components include Notch receptors, Notch ligands, and CSL-DNA-binding proteins. In mammals, there are four receptors (Notch1–4) and five ligands (Jagged1, Jagged2, DLL1, DLL3, DLL4), which initiate signaling upon direct cell–cell contact [34]. Functionally, Notch signaling participates in vascular remodeling and arteriovenous specification by regulating tip/stalk cell differentiation, endothelial proliferation, and vascular homeostasis. Notably, deficiency or mutation of key genes such as Notch1, DLL4, and Jagged1 leads to severe vascular malformations, rupture, and developmental defects, further confirming the pivotal regulatory role of this pathway in angiogenesis [35]. Studies in IH reveal that Notch3 is highly expressed in HemSCs, whereas during differentiation, Notch3 decreases while Notch1/4/Jagged1 increase, driving HemSCs to commit toward HemEC differentiation and forming the aberrant vasculature characteristic of IH [36]. In addition to regulating angiogenesis, Notch is also involved in cell fate switching. Xu et al. [37] demonstrated that pharmacological inhibition of Notch with DAPT upregulates adipogenic key factors such as C/EBP and PPAR-γ, while promoting HemSC proliferation, apoptosis, migration, and lipid accumulation, thereby potentially accelerating IH involution. In summary, Notch signaling not only drives pathological IH angiogenesis by regulating HemEC differentiation and vascular morphogenesis but also promotes tumor regression upon its inhibition by inducing HemSC adipogenic conversion.

#### 3.2.4. Renin–Angiotensin System (RAS)

The RAS has been established as a critical regulatory system in the pathogenesis of IH, and its components and functions have been confirmed by multiple studies. The RAS comprises a classical axis and an alternative axis. The classical axis consists of angiotensin-converting enzyme (ACE), angiotensin II (ATII), and ATII receptors type 1 and type 2 (AT1R and AT2R). The alternative axis is composed of ACE2, angiotensin-(1-7), and the Mas receptor [38]. In IH tissues, local RAS components including ACE, AT2R, and the (pro)renin receptor (PRR) are expressed. PRR is localized to both the CD34-positive endothelial cell population and the CD34-negative non-endothelial cell population [39]. At the functional level, ATII drives IH cell proliferation through AT2R. PRR serves as a component of the Wnt signaling receptor complex. Exogenous renin can promote IH endothelial cell proliferation through PRR by activating the Wnt/beta-catenin pathway, and this effect can be reversed by the Wnt blocker Dickkopf-1 (DKK1) [40]. In addition, various triggering factors may simultaneously upregulate hypoxia-inducible factor-1alpha (HIF-1alpha), which is downstream of the RAS, through angiotensin II [41]. In terms of treatment, modulation of the RAS has become an important strategy for IH management. Propranolol, as a first-line therapeutic agent, reduces serum levels of renin, ACE, and ATII [42]. Captopril, an ACE inhibitor, also lowers ATII levels and induces accelerated involution of IH. A randomized controlled trial confirmed that propranolol is superior to captopril in clinical efficacy, speed of onset, and safety profile [43]. Notably, multiple studies support the role of the RAS in regulating stem cells [44]. Furthermore, the expression of PRR in IH colocalizes with stem cell markers OCT4 and NANOG, suggesting that the RAS may participate in IH pathogenesis by influencing the biological behavior of stem cells. In addition, bypass pathways of the RAS, including cathepsins B, D, and G and chymase, may circumvent classical RAS blockade and sustain ATII production. This provides a potential explanation for the variable response of IH to beta-blocker therapy. Collectively, these findings indicate that the RAS and its bypass pathways constitute an important regulatory network in the pathogenesis of IH. Targeting the RAS and its related pathways provides a theoretical foundation and potential strategies for the treatment of IH.

In IH, several additional pathways cooperatively participate in angiogenesis and disease modulation. In the FGF/FGFR pathway, FGF2 is significantly overexpressed during the proliferation phase and promotes HemEC migration through activation of ERK1/2 phosphorylation [45]. In contrast, the PDGF/PDGFR pathway exhibits complex bidirectional regulation in IH. PDGF-B is elevated in proliferation and, via PDGFR-β signaling, suppresses HemSC adipogenic differentiation, thus maintaining the vascular proliferative phenotype. The negative regulatory role of the PDGF-BB/PDGFR-β signaling pathway in IH involution was further validated by a series of experiments, including tissue expression analysis, stem cell culture, ligand treatment, receptor inhibition, and knockdown [16]. Additionally, the IGF pathway also exerts dual effects in IH. IGF-2 is highly expressed during proliferation and activates the PI3K/AKT pathway via IGF-1R, promoting HemSC proliferation and adipogenic differentiation [46], whereas miR-139-5p targeting of IGF-1R attenuates HemSC proliferation, migration, and adipogenic capacity [47].

### 3.3. Non-Coding RNA and Epigenetic Regulatory Networks

Recent studies have unveiled important regulatory roles of non-coding RNAs in IH angiogenesis. In terms of the miRNA regulatory network, a meta-analysis identified miR-9, miR-939, and the let-7 family as the miRNAs that most extensively interact with dysregulated genes in IH. Further studies revealed that upregulation of miR-15a and downregulation of miR-205 jointly target AKT3 and AKT1, leading to a collective increase in total AKT activity, which in turn promotes HemEC proliferation [48]. In addition, miR-424 targets FGFR1 and miR-206 targets VEGFA, suppressing HemEC proliferation, migration, and tube formation. Beyond the extensive miRNA regulation, lncRNAs deeply participate in IH progression through competitive endogenous RNA mechanisms [49]. NEAT1 has been found to be upregulated in IH, and it exerts its effects through two distinct mechanisms. On one hand, it binds to and stabilizes LDHB, thereby enhancing H3K18 lactylation, which transcriptionally activates β-catenin [6]. On the other hand, it acts as a sponge for miR-361-5p, leading to upregulation of VEGFA [50]. Additionally, Li et al. [51] further revealed that MALAT1 activates the MEKK3/NF-κB pathway by binding to miR-424. Notably, Wang et al. [52] demonstrated that propranolol can downregulate NEAT1, thereby relieving its suppression of miR-194-5p and consequently inhibiting TRAF6/NF-κB-mediated angiogenesis. Taken together, these findings suggest that targeting lncRNAs may represent a potential therapeutic strategy for IH. Beyond the regulation by miRNAs and lncRNAs, m6A epitranscriptomic modifications also play a critical role in IH. The E3 ubiquitin ligase HECW2 is upregulated in IH, and it modulates the ubiquitination of ALKBH5, which in turn enhances the m6A demethylation of LDHA mRNA. This leads to upregulated LDHA expression and increased glycolysis, thereby promoting IH progression. In addition, ALKBH5 upregulates FOXF1 in an m6A-YTHDF2-dependent manner, which subsequently activates HK-2 signaling and promotes malignant behaviors of IH cells [53].

During IH involution, a variety of cytokines and immune cells cooperatively participate in the regulatory process. Screening for anti-angiogenic factors has revealed that PEDF is markedly upregulated in involuting HemECs, where it acts in an autocrine manner to suppress HemEC growth, thereby accelerating vascular regression. At the immune cell level, infiltration of M1 macrophages has been confirmed to increase during involution. Specifically, these macrophages secrete cytokines such as tumor necrosis factor-α (TNF-α) to induce EndMT in HemECs, a process characterized by downregulation of endothelial markers and upregulation of mesenchymal markers, which in turn promotes vascular regression and conversion to fibrofatty tissue. Meanwhile, Yue et al. [54] further demonstrated that M1 macrophage-derived exosomes can also induce EndMT and suppress HemEC proliferation through activation of the AMPK/SIRT1/PGC1α signaling pathway. In addition, mast cells (MCs) are actively involved in the involution process. Hou et al. [55] showed that estrogen receptor α-positive MCs are significantly increased in number during involution, and their activation may release regulatory factors that contribute to vascular regression. At the level of stem cell fate switching, KIAA1429 has been confirmed to be highly expressed in involuting IH, where it promotes regression by enhancing HemSC stemness and inducing transdifferentiation of HemECs toward a fibroblast-like phenotype. Additionally, miR-139-5p was found to inhibit HemSC proliferation and migration while promoting their adipogenic differentiation by targeting IGF-1R, thereby providing a molecular explanation for adipose tissue formation during involution. Collectively, these mechanisms constitute a complex regulatory network driving the transition from proliferation to involution in IH, wherein non-coding RNAs and epigenetic modifications play pivotal roles in determining cell fate.

## 4. Factors Influencing Angiogenesis and Involution

**Figure 3:**
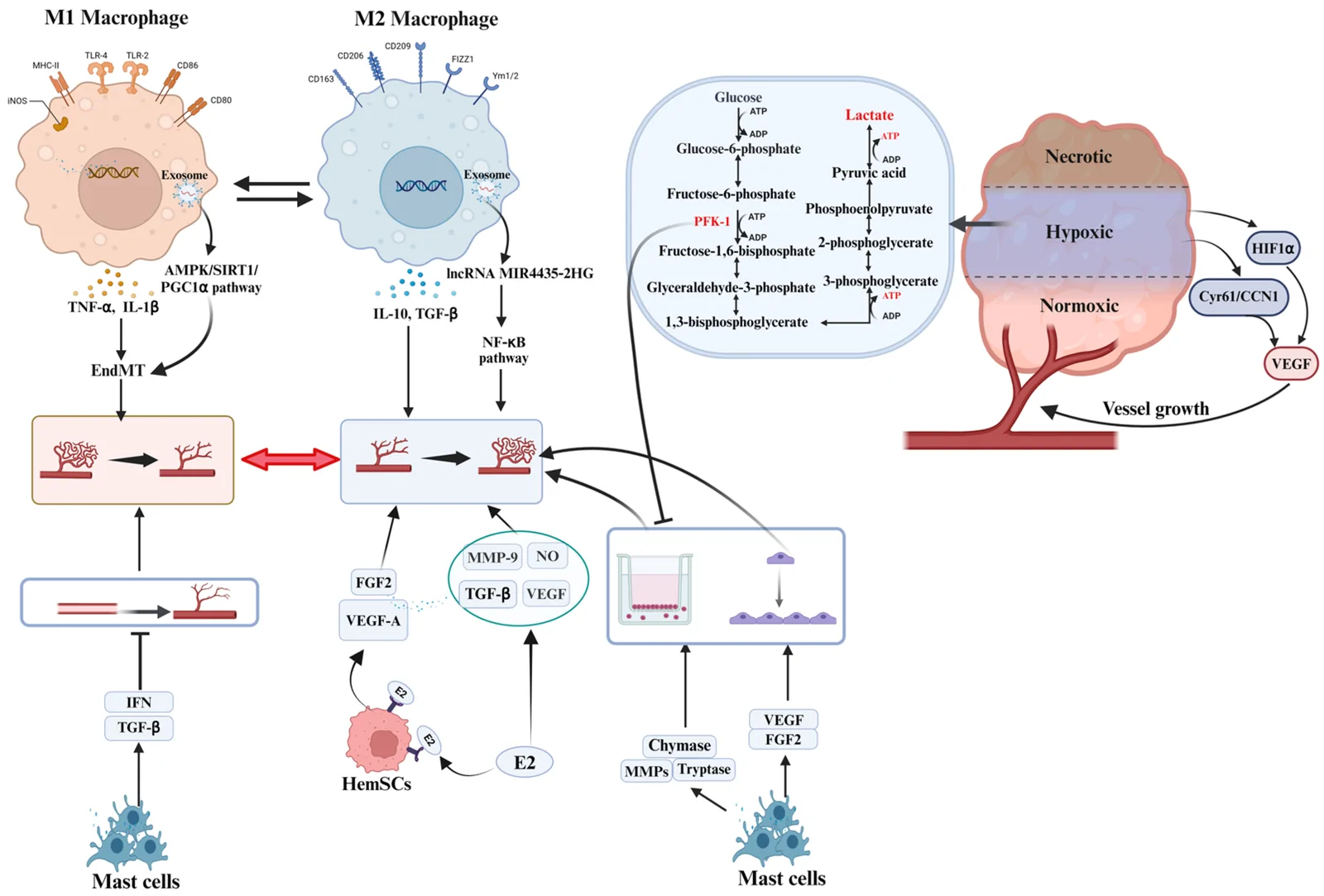
Key factors regulating angiogenesis and involution. Created in BioRender. Wu, Y. (2026) https://BioRender.com/3svs9j7.

Beyond cell-intrinsic mechanisms, a variety of extrinsic microenvironmental and systemic factors profoundly modulate the angiogenic and involutive processes of IH by regulating the aforementioned signaling pathways (Figure 3). Among these, estrogen is regarded as a key systemic regulator, closely linked to the higher prevalence in female infants [56]. Meanwhile, the hypoxic microenvironment drives VEGF expression through stabilization of HIF-1α, thereby promoting neovascularization during proliferation [57,58]. In terms of the immune microenvironment, infiltration of M1 macrophages and MCs increases during involution, and these cells induce EndMT via secretion of cytokines or exosomes, consequently facilitating vascular regression. In addition, other factors such as insulin-like growth factor, pigment epithelium-derived factor, and various chemokines are also involved in regulating cell proliferation, differentiation, and involution [59]. These factors act either synergistically or antagonistically to collectively form an external regulatory network for IH. The following sections will elaborate on estrogen regulation, hypoxia and metabolic remodeling, immune microenvironment modulation, and other regulatory factors in detail.

### 4.1. Estrogen Regulation

The marked female predominance of IH suggests a critical role for estrogen in its pathogenesis. The major physiological estrogens in humans include estrone (E1), estradiol (E2), and estriol (E3), among which E2 possesses the highest biological activity [60]. Multiple studies have confirmed that serum estrogen concentrations are significantly elevated in children with proliferating IH compared with healthy controls, whereas they decline markedly during involution. Concurrently, high expression levels of estrogen receptors such as ER-α, ER-β, and PR are detected in IH tissues [61]. Zhang et al. [62] directly demonstrated through in vitro and in vivo experiments that E2 binding to ER-α on HemSCs upregulates VEGF-A and FGF2 expression, thereby promoting angiogenesis and IH growth. Sun et al. further hypothesized that estrogen may influence IH formation by modulating key pro-angiogenic factors including MMP-9, endothelial progenitor cells (EPCs), VEGF, and NO. Meanwhile, linking estrogen to immune cells, it was found that ER-α is expressed on MCs but not on endothelial cells in IH, and that the number of E2-positive MCs increases significantly as IH progresses from proliferation to involution. This observation suggests that the effects of estrogen may be partially mediated through MCs—promoting angiogenesis during proliferation and possibly cooperating with MCs to initiate involution. Additionally, estrogen can regulate the secretion of multiple pro- and anti-angiogenic factors such as TGF-β, indirectly modulating tumor angiogenesis and regression. Furthermore, studies on breast, ovarian, and uterine cancers also support an association between estrogen and angiogenic activity [63,64]. Nevertheless, the precise mechanisms by which estrogen regulates IH remain incompletely elucidated, and its effects on intracellular signaling pathways are still debated, warranting further systematic investigation.

### 4.2. Hypoxia and Metabolic Remodeling

The hypoxic microenvironment is a critical driver of IH angiogenesis and progression, exerting regulatory effects throughout the entire course of IH development through complex multi-molecular and multi-pathway mechanisms. Hypoxia-inducible factors (HIFs) are key transcription factors mediating cellular responses to hypoxia. Under hypoxic conditions, degradation of HIF-1α or HIF-2α is impeded, leading to their intracellular accumulation [65], which in turn activates downstream genes such as GLUT-1 and VEGF, accelerating HemEC proliferation and neovascularization [66,67]. Some studies have suggested that the hypoxic environment in IH may be primarily regulated by HIF-2α rather than the classic HIF-1α. Nevertheless, a substantial body of evidence still supports a major role for HIF-1α in IH. Current data show that HIF-1α levels in IH tissues are significantly higher than in normal vascular tissues, and that propranolol treatment reduces HIF-1α expression in a time- and dose-dependent manner. Zhang et al. [68] further clarified that HIF-1α modulates the cell cycle of HemECs by upregulating VEGF/VEGFR-2 expression, which promotes Cyclin D1 and suppresses p53, thereby stimulating proliferation and inhibiting apoptosis. It has been confirmed that propranolol acts mainly via β2-AR to suppress VEGF and STAT3 signaling in a HIF-1α-dependent manner, and that HIF-1α overexpression significantly reverses the therapeutic effects of propranolol. In addition, hypoxia markedly upregulates cysteine-rich angiogenic inducer 61 (Cyr61/CCN1), which promotes VEGF-A production, accelerates downstream angiogenic factor expression and activity in HemSCs, and stimulates HemSCs-to-HemECs transition, forming a positive feedback loop [69]. Beyond its pro-angiogenic effects, hypoxia is closely linked to metabolic reprogramming in IH cells. Hypoxia enhances glycolysis to supply energy for angiogenesis, and this glycolytic enhancement can be partially reversed by inhibiting HIF-1α [70]. Using microarray analysis, Yang et al. [71] found that multiple metabolism-related pathways, such as oxidative phosphorylation and glycogen biosynthesis, are significantly altered in proliferating IH. Furthermore, they demonstrated that inhibition of the glycolytic key enzyme PFK-1 suppresses HemEC proliferation and migration, while also reducing glucose uptake as well as lactate and ATP production. Further studies have revealed a novel epigenetic mechanism of lactylation modification, in which NEAT1 stabilizes LDHB to increase intracellular lactate levels, which in turn enhances H3K18 lactylation at the β-catenin promoter region and transcriptionally activates pro-proliferative signals. Based on the above findings, an inferred regulatory model can be proposed. In this model, hypoxia-activated HIF-1α may drive metabolic reprogramming and upregulate glycolysis, thereby promoting lactate accumulation and histone lactylation, which in turn participates in the transcriptional regulation of pro-angiogenic genes. The validity of this model remains to be established through systematic experimental verification in future studies.

### 4.3. Immune Microenvironment Regulation

Immune cells exert distinctly different regulatory functions at various stages of IH, serving as critical extrinsic drivers for the transition from angiogenesis to involution. The coordinated actions of inflammatory cytokines and immune cells collectively form a bidirectional regulatory network governing IH angiogenesis. Among these, the dynamic balance between M1 and M2 macrophages is particularly crucial. During IH involution, M1-polarized macrophages exhibit prominent perivascular infiltration and, through secretion of cytokines such as TNF-α and IL-1β, induce EndMT in HemECs, leading to downregulation of endothelial markers including CD31 and upregulation of mesenchymal markers such as Snail and Slug. Importantly, HemECs treated with M1 macrophages can differentiate into adipocytes under adipogenic induction, partially recapitulating the natural course of IH involution in vitro. Furthermore, it has been demonstrated that exosomes derived from M1 macrophages can similarly induce EndMT and suppress HemEC proliferation through activation of the AMPK/SIRT1/PGC1α signaling pathway. In contrast, during the proliferative phase of IH, M2 macrophages predominate and mediate immune evasion by secreting IL-10 and TGF-β [14,72]. Moreover, exosomes derived from M2 macrophages can deliver the lncRNA MIR4435-2HG, which binds to and regulates HNRNPA1, thereby activating the NF-κB pathway and promoting IH progression [73]. Additionally, proliferating IH exhibits high local concentrations of inflammatory cytokines, which upregulate VEGFA expression through JAK/STAT and NF-κB signaling, amplifying aberrant angiogenesis [74,75]. Beyond macrophages, MCs also participate bidirectionally in IH progression. Xia et al. [76] reviewed the role of MCs in the IH life cycle, suggesting that E2 may act as a chemotactic factor attracting MCs to migrate into IH and become activated. Activated MCs secrete either pro- or anti-angiogenic regulators at different stages, and imbalance of these factors may contribute to IH proliferation and involution. Clinical samples confirmed that ER-α and E2 are expressed on MCs rather than on IH endothelial cells, and that the numbers of E2-positive and tryptase-positive MCs are significantly greater in involuting IH than in proliferating IH, further supporting the involvement of estrogen in IH involution via MCs. In addition to immune cells, certain cytokines also exhibit bidirectional regulatory properties. For example, TNF-α tends to promote angiogenesis when HemPCs coverage is high and uniform, but inhibits angiogenesis when coverage is low [77]. Allograft inflammatory factor-1 (AIF-1) is highly expressed in some IH endothelial cells and can recruit myeloid cells to form an inflammatory microenvironment, although its cellular origin remains unclear [78]. In summary, immune cells along with their secreted cytokines and exosomes profoundly influence the transition from proliferation to involution through dynamic balance and bidirectional regulation.

### 4.4. Other Regulatory Factors

In addition to the hormonal, hypoxic/metabolic, and immune-related mechanisms described above, various cell-of-origin factors, cell-cycle regulators, and signaling molecules also play important roles in IH angiogenesis and involution. Jia et al. [79] proposed the hypothesis that monocytes may serve as a potential source of HemECs. Their study found abundant monocytes in IH, and monocytes are precursors of endothelial progenitor cells. Although the number of EPCs is small, monocytes may home to IH and differentiate toward an endothelial lineage, directly participating in early vascular expansion. Meanwhile, MCs, as another major immune cell population in IH tissue, are present throughout the entire IH developmental cycle. Studies have shown a positive correlation between MC density and microvessel density, and MCs migrate into IH via the circulation and become activated. Activated MCs predominantly secrete VEGF and FGF2 to promote HemEC proliferation during the proliferative phase and strongly express multiple bioactive factors that collectively function during IH development. MCs also release chymase and tryptase, which are proangiogenic modulators that facilitate HemECs migration and contribute to vascular growth [55,80]. However, other studies have indicated that MCs secrete anti-angiogenic factors such as IFN and TGF-β [76,81], which inhibit HemEC proliferation and microvessel differentiation, and induce mesenchymal cells to transdifferentiate into HemPCs. The role of MCs in IH progression is complex, and whether they specifically promote or inhibit angiogenesis may depend on the conditions of the stromal microenvironment. Concurrently, the roles of cell-cycle regulators have drawn attention. Wang et al. [82] found by immunohistochemistry that p16 protein expression is significantly lower in proliferating IH than in involuting IH, and remains lower than in normal tissue, suggesting that p16 may participate in IH involution by inhibiting angiogenesis and inducing endothelial senescence or apoptosis. Moreover, multiple secreted factors regulate IH cell fate in an autocrine or paracrine manner. Among them, PEDF is upregulated in involuting HemECs and accelerates IH regression by inhibiting HemEC growth. In contrast, PDGF is highly expressed during proliferation and inhibits HemSC adipogenic differentiation via PDGF receptor β signaling, thus negatively regulating involution. Furthermore, molecules such as PDK1 [83], STC2, and USP18 have also been confirmed to participate in regulating HemEC proliferation and angiogenesis. Collectively, these findings further enrich the regulatory network governing IH angiogenesis and involution.

## 5. Regulation of Angiogenesis and Involution by Bioactive Components of Danshen

**Figure 4:**
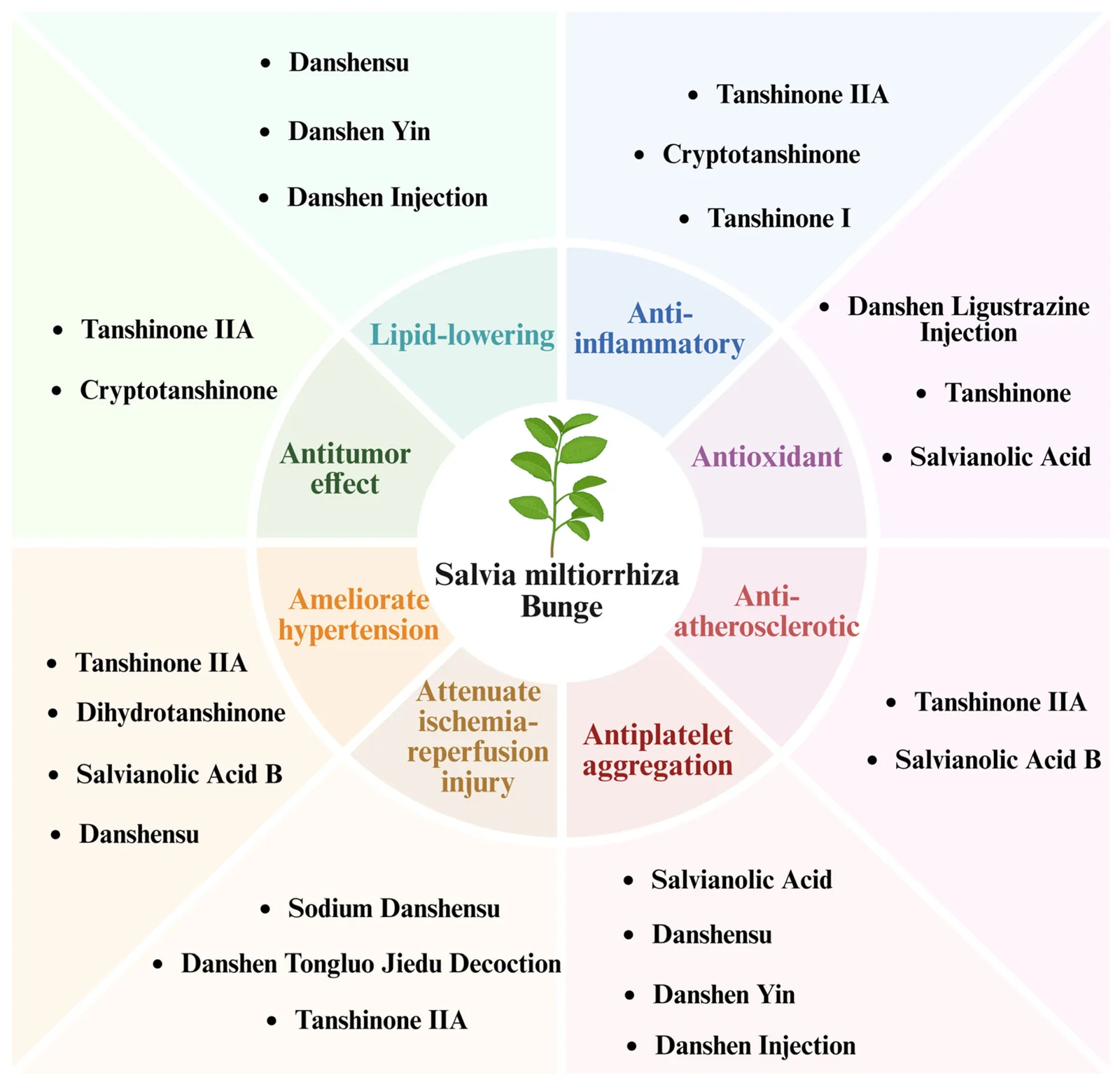
Major pharmacological effects of Danshen and its bioactive constituents. Created in BioRender. Lu, Y. (2026) https://BioRender.com/7228yv9.

Danshen is a medicinal herb widely used in traditional Chinese medicine. In classical Chinese medical theory, it is categorized as a blood-activating and stasis-resolving agent, and is commonly employed in the treatment of cardiovascular diseases, cerebrovascular disorders, and neoplastic diseases, among others (Figure 4) [84]. The plant contains a variety of bioactive constituents, among which the lipophilic tanshinones and hydrophilic salvianolic acids are the most prominent [85,86]. Recent studies have demonstrated that these bioactive constituents can regulate angiogenesis and involution at multiple levels. On one hand, they suppress pathological angiogenesis and promote regression of aberrant vessels through mechanisms such as inhibiting VEGF/COX-2 signaling, inducing apoptosis, and arresting the cell cycle, making them suitable for treating hyperangiogenic diseases including tumors and hemangiomas. On the other hand, they stimulate physiological angiogenesis by activating pathways such as VEGF/VEGFR signaling and the PI3K/AKT/mTOR axis, thereby promoting vascular repair in ischemic conditions such as myocardial and cerebral ischemia (Table 1). This bidirectional regulatory property positions the active components of Danshen as highly promising candidates for the treatment of angiogenesis-related disorders.

### 5.1. Tanshinone IIA

Tanshinone IIA is an important lipophilic component of Danshen. It can synergistically regulate pathological angiogenesis in IH through multiple pathways, suppressing lesion proliferation and promoting involution, thus representing a potential natural anti-hemangioma agent. In human umbilical vein endothelial cells (HUVECs), tanshinone IIA has been shown to partially inhibit endothelial cell proliferation, migration, and tube formation by suppressing the VEGF/VEGFR2 signaling axis and reducing MMP-2/-9 secretion [87]. Beyond these endothelial cell-based findings, evidence from other tumor models further supports the anti-angiogenic potential of tanshinone compounds. In a gastric cancer model, diterpenoid tanshinones, which are a group of active components from Danshen, significantly reduced the expression of phosphorylated PI3K, AKT, and mTOR proteins in SGC-7901 gastric cancer cells and tumor tissues, consequently downregulating VEGF levels and inhibiting tumor growth [88]. In addition, Zhou et al. [89] found that tanshinone IIA suppresses HUVEC proliferation, tube formation, and migration in a dose-dependent manner by decreasing the expression of HIF-1α, VEGF, and bFGF in HCT-116 cells, thereby reducing colorectal cancer angiogenesis. Beyond these mechanisms, tanshinone IIA also inhibits aberrant proliferation and phenotypic switching of vascular smooth muscle cells. In vascular smooth muscle cells and a mouse carotid artery ligation model, tanshinone IIA was reported to attenuate neointima formation induced by vascular injury and promote the transition of VSMCs from a synthetic to a contractile phenotype via upregulation of KLF4, thereby mitigating pathological vascular remodeling [90]. Additionally, tanshinone IIA suppresses tumor angiogenesis in colorectal cancer through downregulation of COX-2 and VEGF expression [91]. The aforementioned evidence is predominantly derived from tumor and vascular injury models, and direct validation in IH-specific cellular or animal models remains lacking. Nevertheless, a recent pilot clinical study has provided direct evidence for the clinical application of tanshinone in IH. This study enrolled 29 infants with superficial IH, who received topical tanshinone compounds three times daily. After 6 months of treatment, the skin erythema index was significantly reduced (*p* < 0.001), with 79.31% of parents reporting satisfaction with the therapeutic response, and no serious adverse events were documented. This study validated the safety and potential efficacy of topical tanshinone in IH patients. However, its single-arm design and relatively small sample size limit the generalizability of the conclusions, necessitating further confirmation through large-scale randomized controlled trials [92]. Collectively, these findings indicate that tanshinone IIA not only acts directly on endothelial cells but also synergistically inhibits pathological angiogenesis by modulating the functions of smooth muscle cells and other stromal cells.

### 5.2. Dihydrotanshinone I (DHTS)

In addition to tanshinone IIA, DHTS is another important lipophilic active component of Danshen. Studies have shown that this natural compound inhibits angiogenesis both in vitro and in vivo [93], with its action involving key pathways such as VEGF/VEGFR2, HIF-1α/HuR, and MMP9, through inducing hemangioma cell apoptosis and blocking aberrant angiogenesis. Cai et al. [94] utilized the murine EOMA hemangioendothelioma cell line and the corresponding EOMA xenograft models to screen 14 major compounds from Danshen, and identified DHTS as the most potent in modulating the biological behaviors of hemangioma cells. In that study, in vitro experiments on EOMA cells showed that DHTS induced apoptosis with significantly higher efficiency than propranolol, the first-line drug for IH, within this cellular model. Furthermore, experiments in nude mouse xenograft models bearing EOMA tumors demonstrated that 10 mg/kg of DHTS significantly suppressed tumor growth, with efficacy comparable to that of 40 mg/kg propranolol in these animal models. Further mechanistic exploration revealed that DHTS reduced hemangioma volume by markedly downregulating the protein expression of VEGFR2 and MMP9. Immunohistochemical results also confirmed that after DHTS intervention, the expression of CD34, VEGFR2, and MMP9 in hemangioma tissues was significantly inhibited, while the expression of the pro-apoptotic factor Caspase-3 was markedly increased, thereby suppressing aberrant angiogenesis and inducing apoptosis of hemangioma cells. Moreover, Duan et al. [95] proposed that DHTS can interfere with the binding of the RNA-binding protein HuR to HIF-1α mRNA, thereby blocking the post-transcriptional processing of HIF-1α, and consequently targeting the HIF-1α signaling pathway to inhibit hemangioma cell proliferation and angiogenesis. However, pharmacokinetic data in juvenile animal models and safety profiles in the infant population remain unclear, representing a critical gap that must be addressed prior to clinical translation.

### 5.3. Salvianolic Acid B (SalB)

SalB is a core hydrophilic active ingredient of Danshen. Current evidence indicates that SalB exhibits complex, bidirectional regulatory effects on angiogenesis, with its action depending on the microenvironmental context. In tumor and inflammatory microenvironments, SalB predominantly suppresses aberrant angiogenesis and modulates endothelial cell proliferation and apoptosis. The underlying mechanisms primarily involve targeted regulation of multiple signaling pathways such as VEGF/COX-2, through which SalB intervenes in angiogenesis at several levels, including inhibition of hypoxic microenvironment activation, reduction in VEGF secretion, and blockade of endothelial cell proliferation [96,97]. For instance, Zhang et al. [98] reported that SalB significantly reduces the viability, invasion, and migration of HeLa cervical cancer cells and induces apoptosis via suppression of the PI3K/AKT pathway, suggesting a similar inhibitory effect on tumor angiogenesis. In contrast, under ischemic or injurious conditions, SalB promotes functional vascular repair. Tang et al. [99] found that SalB significantly enhances the migration and tube-forming capacity of human bone marrow-derived endothelial progenitor cells, and attenuates oxidative stress-induced damage by activating the AKT/mTOR/4EBP1 pathway while inhibiting p38 MAPK/ATF2 and ERK1/2 signaling, indicating its potential pro-angiogenic role in ischemic diseases. Notably, when combined with other active components, SalB exhibits enhanced angiogenic effects. Chen et al. [100] demonstrated that co-administration of SalB and ferulic acid synergistically promotes intersegmental vessel sprouting in zebrafish and G_1_-to-S phase progression in HUVECs by upregulating VEGFR2 and its ligands VEGF-A and VEGF-B, thereby exerting pro-angiogenic activity. Collectively, these studies indicate that the regulation of angiogenesis by SalB is context-dependent, primarily suppressing pathological angiogenesis in tumor and inflammatory microenvironments while promoting functional vascular repair under ischemic or damaged conditions. Given that angiogenesis during the proliferative phase of IH shares mechanistic similarities with tumor angiogenesis, the anti-angiogenic effect of SalB in the tumor microenvironment suggests that it may have potential therapeutic value for pathological angiogenesis in IH.

### 5.4. Other Bioactive Components

Danshensu (DSS) is another important water-soluble active constituent of Danshen, exhibiting multi-target and multi-pathway pharmacological activities, with demonstrated potential in renal protection, anti-inflammation, antiviral therapy, and combined cancer treatment [101,102,103,104]. Using mouse tumor models and hypoxic endothelial cell assays, combined with molecular target validation, Qian et al. [105] utilized LLC and B16F10 subcutaneous allograft models along with hypoxia-induced HUVEC assays to demonstrate that DSS can inhibit endothelial glycolysis by targeting PKM2 and activate the β-catenin/Claudin-5 signaling axis to enhance tight junctions, thereby inducing tumor vascular normalization and improving chemotherapeutic drug delivery. In addition to DSS, cryptotanshinone (CPT) is another major lipophilic diterpenoid compound found in Danshen [106]. Studies have shown that CPT possesses clear anti-angiogenic and anti-tumor activities. Xu et al. [107] confirmed that CPT significantly inhibits VEGF-induced proliferation, migration, invasion, and tube formation of human umbilical vein endothelial cells, with the mechanism closely related to the suppression of VEGFR2 phosphorylation and its downstream Src/FAK and ERK1/2 signaling pathways. Under hypoxic conditions, CPT also downregulates the expression of HIF-1α and AEG-1 in PC-3 prostate cancer cells, reduces VEGF secretion, and interferes with endothelial tube formation [108]. Furthermore, CPT exhibits cardioprotective effects, reverses multidrug resistance, and sensitizes combination therapy, involving pathways such as STAT3 and NF-κB [109]. Collectively, these findings indicate that CPT is a multi-targeted active component with potential value in inhibiting pathological angiogenesis and tumor growth.

**Table 1:** Regulatory effects and mechanisms of Danshen bioactive constituents on angiogenesis and involution.

Bioactive Constituents	Chemical Structures	Major Models	Core Signaling Pathways	Working Concentration Range	Major Mechanisms of Action	References
Tanshinone IIA	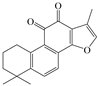	Chick embryo chorioallantoic membrane (CAM) and rat aortic ring assay	VEGF/VEGFR2	4–12 μM	VEGFR2/CD146 ↓; endothelial proliferation, migration, tube formation ↓; MMP-2/9 secretion ↓ (mRNA unchanged); ECM degradation ↓; angiogenesis ↓	[87]
Dihydrotanshinone I	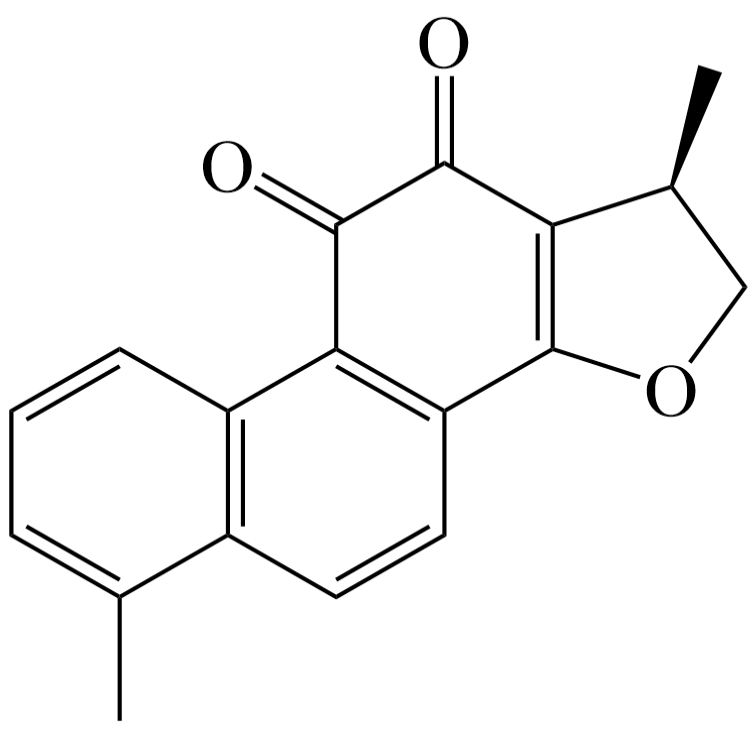	CAM (*n* = 10)	Not identified	0.25, 0.5, 1 μg/mL (HUVECs); 0.1, 0.2 μg/egg (CAM)	Endothelial proliferation, migration, invasion, tube formation ↓; angiogenesis ↓	[93]
		BALB/c nude mice with subcutaneous EOMA xenografts (1 × 10^7^ cells/mouse) in the axilla.	Mitochondrial apoptosis pathway; Fas-mediated apoptosis pathway; VEGFR2/MMP-9-mediated angiogenesis pathway	10 mg/kg	Low concentration: Bax/PARP/AIF/Caspase9/Caspase3/Cyt-c ↑ (mitochondrial pathway); High concentration: FADD/Caspase8 ↑ (Fas pathway); VEGFR2/MMP-9 ↓; tube formation ↓; angiogenesis ↓	[94]
Salvianolic acid B	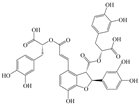	① ICR mouse sponge implantation model (*n* = 6); ② flk-1:GFP zebrafish embryos (*n* = 48)	IRE1/ASK1/JNK/p38MAPK; VEGF	2.7, 8.3, 25 μM	VEGF ↓; AngII-induced angiogenesis ↓ (type I IRE1 kinase inhibitor, binds Lys599/Lys690)	[96]
		Female Swiss albino mice with ESC subcutaneous xenograft (*n* = 10/group)	VEGF/COX-2	25 mg/kg	VEGF ↓; COX-2 ↓; MMP-9 ↓; Cyclin D1 ↓; angiogenesis ↓	[97]
Danshensu	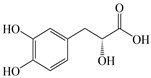	C57BL/6J mice with subcutaneous LLC/B16F10 tumors (*n* = 5/group)	PKM2 (Lys433); β-Catenin/Claudin-5; Keap1/Nrf2/HO-1/NQO1	40 mg/kg	PKM2 ↓ (glycolysis ↓); β-Catenin/Claudin-5 ↑; tight junctions ↑; vascular permeability ↓; vascular normalization ↑; tumor hypoxia ↓	[105]
Cryptotanshinone	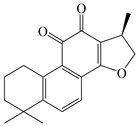	Not performed (in vitro only, HUVECs)	VEGFR2 (Tyr1175); Src/FAK; ERK1/2/p90RSK	5, 10, 20 μmol/L (HUVEC)	VEGFR2 Tyr1175 phosphorylation ↓; Src/FAK ↓; ERK1/2/p90RSK ↓; endothelial proliferation, migration, invasion, tube formation ↓; angiogenesis ↓	[107]
		BALB/c nude mouse PC-3 xenograft model (*n* = 5/group)	PI3K/AKT	10 mg/kg	HIF-1α ↓; AEG-1 ↓; VEGF ↓; HIF-1α binding to VEGF promoter ↓; endothelial tube formation ↓; angiogenesis ↓	[108]

↓ indicates decrease; ↑ indicates increase.

## 6. Conclusions and Perspectives

IH, as a common benign vascular tumor of infancy, is characterized by a unique natural course of proliferation followed by spontaneous involution, which fundamentally reflects a pathological imbalance between angiogenesis and regression. Current studies suggest that the multilineage differentiation fate switching of HemSCs is considered to be one of the important cellular bases driving disease progression, while the networked activation and inhibition of core signaling pathways such as VEGF/VEGFR, PI3K/AKT/mTOR, and Notch form the molecular regulatory hub that governs aberrant angiogenesis and spontaneous involution. Epigenetic mechanisms such as non-coding RNAs, m6A methylation, and lactylation modifications further fine-tune the expression of key genes at both transcriptional and post-transcriptional levels, thereby participating in endothelial cell proliferation, apoptosis, and transdifferentiation. Concurrently, extrinsic factors including estrogen, hypoxic microenvironment, M1/M2 macrophage polarization, and immune microenvironment remodeling interact with intracellular signaling networks to collectively determine the initiation, progression, and outcome of IH.

However, it must be critically pointed out that nearly all current conclusions regarding the mechanisms of IH are derived from in vitro experiments on hemangioma endothelial cells and stem cells, as well as from small-sample animal xenograft models. High-quality prospective human clinical validations and large-cohort multi-omics evidence remain extremely scarce. More critically, the drug concentrations and dosing regimens used in most in vitro experiments are higher than the clinically achievable safe exposure levels, and animal models (such as EOMA mouse xenografts) are fundamentally different from infantile IH in terms of immune microenvironment, tissue architecture, and growth kinetics, rendering the translational extrapolation from “effective in cells/animals” to “benefit in infants” highly uncertain and risky. Conducting an in-depth critical analysis of the angiogenesis and involution mechanisms of IH, and distinguishing the scientific questions that can be explained by existing preclinical data from the unresolved key gaps, can provide innovative ideas for the basic biological research and clinical diagnosis and treatment of IH. This multi-dimensional and multi-layered understanding of the regulatory mechanisms provides important theoretical support for elucidating the biological essence of the unique life cycle of IH.

Traditional Chinese medicine possesses distinct advantages in the syndrome-based differentiation and treatment of vascular-related disorders. As a representative herb for promoting blood circulation and resolving stasis, Danshen has been extensively validated for its bidirectional regulatory effects on angiogenesis. Its core bioactive constituents, including tanshinone IIA, DHTS, SalB, and cryptotanshinone, exert significant anti-pathological angiogenesis effects through multiple mechanisms, such as targeted inhibition of pro-angiogenic pathways, downregulation of HIF-1α expression, induction of aberrant endothelial cell apoptosis, and blockade of matrix degradation and vascular remodeling.

Despite the growing body of research on IH pathogenesis and the intervention of Danshen components, several limitations remain. Most current studies are confined to individual signaling pathways or single active ingredients, lacking a systematic analysis of pathway cross-talk, cellular microenvironment interactions, and molecular network synergy. Aside from tanshinone gel, the anti-hemangioma effects of other active components derived from Danshen have only been verified in cellular experiments in vitro and in animal models. However, the hepatic and renal functions, along with the metabolic enzyme systems, are not yet fully matured in infants, which may result in pharmacokinetic profiles that differ by orders of magnitude between adults and this pediatric population. Furthermore, the dosing regimens adopted in all existing animal studies fail to recapitulate the actual clinical administration scenarios in IH patients, thereby constituting a marked inadequacy in clinical translation. Moreover, the synergistic mechanisms of multiple Danshen components and their potential for enhancing efficacy and reducing toxicity when combined with propranolol warrant further investigation.

Future research should leverage cutting-edge technologies such as multi-omics and molecular targeting to systematically dissect the regulatory networks of IH angiogenesis, elucidate the intrinsic connections between signaling pathways and the local microenvironment, and further define the precise molecular targets of Danshen active ingredients, thereby deepening our understanding of their anti-angiogenic mechanisms. On this basis, large-scale, well-designed clinical trials should be conducted to establish a safe medication system appropriate for the infant population. In addition, novel therapeutic models integrating traditional Chinese medicine formulae and combined Chinese-Western medicine approaches should be actively explored, with the goal of developing new formulations featuring higher targeting specificity, better stability, and fewer adverse effects, ultimately providing efficient, safe, and individualized integrated treatment strategies for IH.

## Data Availability

No new data were created or analyzed in this study. Data sharing is not applicable to this article.
